# A Hybrid CNN-LSTM Architecture for Seismic Event Detection Using High-Rate GNSS Velocity Time Series

**DOI:** 10.3390/s26020519

**Published:** 2026-01-13

**Authors:** Deniz Başar, Rahmi Nurhan Çelik

**Affiliations:** Faculty of Civil Engineering, Department of Geomatics Engineering, Istanbul Technical University, ITU Ayazaga Campus, Maslak, Istanbul 34367, Turkey; celikn@itu.edu.tr

**Keywords:** Global Navigation Satellite Systems (GNSS), Long Short-Term Memory (LSTM), Convolutional Neural Network (CNN), Explainable Artificial Intelligence (XAI), Earthquake Early Warning (EEW)

## Abstract

**Highlights:**

This study evaluates a hybrid CNN–LSTM model for seismic event detection from 5 Hz GNSS velocity time series, trained with pseudo-synthetic data and tested on an inde-pendent real GNSS setting.

**What are the main findings?**

- Hybrid CNN–LSTM model outputs were evaluated both at frame-level and event–station-level, and multiple multi-channel decision schemes (vote-based, any-channel, weighted fusion) were reported under both fixed and validation-calibrated operating points.
- On the independent real GNSS test setting, the horizontal components (*H*0/*H*1) show substantially stronger discrimination than the vertical (*UP*) component, with the largest degradation observed on UP under real ambient noise.

**What are the implications of the main findings?**

- Event–station aggregation and fusion/operating-point choice govern missed-detection vs false-alarm trade-off on real data.
- The observed real-test performance drop is consistent with a measurable training–test distribution shift; MMD- and SHAP-based diagnostics help explain changing decision patterns.

**Abstract:**

Global Navigation Satellite Systems (GNSS) have become essential tools in geomatics engineering for precise positioning, cadastral surveys, topographic mapping, and deformation monitoring. Recent advances integrate GNSS with emerging technologies such as artificial intelligence (AI), machine learning (ML), cloud computing, and unmanned aerial systems (UAS), which have greatly improved accuracy, efficiency, and analytical capabilities in managing geospatial big data. In this study, we propose a hybrid Convolutional Neural Network–Long Short Term Memory (CNN-LSTM) architecture for seismic detection using high-rate (5 Hz) GNSS velocity time series. The model is trained on a large synthetic dataset generated by and real high-rate GNSS non-event data. Model performance was evaluated using real event and non-event data through an event-based approach. The results demonstrate that a hybrid deep-learning architecture can provide a reliable framework for seismic detection with high-rate GNSS velocity time series.

## 1. Introduction

Global Navigation Satellite Systems (GNSS) have become integral in geomatics engineering, enabling precise positioning essential for cadastral surveys, topographic mapping, and infrastructure monitoring [1]. Recent advances have transformed GNSS applications, particularly through its integration with disruptive technologies such as artificial intelligence (AI), machine learning (ML), cloud computing, and unmanned aerial systems (UAS). These technologies have significantly enhanced accuracy, efficiency, and analytical capabilities in geospatial big data management [2]. AI and ML, in particular, have transformed GNSS data processing through improved anomaly detection, predictive analytics, and signal processing under challenging conditions. Additionally, cloud-based solutions facilitate scalable data management and collaboration, while UASs equipped with GNSS sensors enhance rapid and detailed spatial data acquisition. Collectively, these advancements are reshaping modern geomatics practices, leading to innovative solutions and methodologies [1,2,3].

Likewise, geomatics applications depend on GNSS to establish accurate reference frames, precisely determine and adjust geodetic networks, and detect subtle Earth movements—often down to just a few millimeters [4,5]. In geodynamics, GNSS offers valuable insights by tracking tectonic plate movements, monitoring fault lines, analyzing volcanic activities, and observing how glaciers influence the Earth’s crust [6,7]. These GNSS data sets, managed internationally through standardized methods, have significantly advanced our understanding of Earth’s dynamic processes and improved our ability to respond to natural hazards [8]. Real-time GNSS applications extend to precision agriculture, autonomous vehicle navigation, and disaster response, highlighting its versatility [9]. Nevertheless, GNSS signals often suffer from interference caused by buildings, obstacles, or signal reflections (multipath effects), posing challenges for traditional positioning methods [9,10].

To address these issues, researchers increasingly employ machine learning (ML) and deep learning (DL), which offer flexible and adaptive solutions to GNSS data analysis [9,11]. Among these innovative techniques, Convolutional Neural Networks (CNNs) stand out due to their exceptional ability to recognize patterns directly within GNSS signals. CNNs efficiently detect issues like multipath interference or localized signal disruptions without the extensive manual adjustments that are traditionally required [9]. As a result, CNN-based approaches are becoming increasingly popular for improving GNSS signal processing, enhancing data quality, reducing interference, and significantly increasing positioning accuracy—especially under challenging environmental conditions.

Recent research has shown how effectively ML and deep learning (DL) techniques can solve a range of GNSS signal problems. For instance, supervised learning methods have successfully corrected multipath-related errors common in urban environments, while CNNs have been applied to classify different kinds of signal interruptions, detect sudden cycle slips in carrier-phase measurements, and monitor overall signal quality [9,12]. Other methods, such as autoencoders, have proven effective in detecting unusual patterns or anomalies in GNSS signals—like unexpected drops in signal strength or interruptions in satellite visibility. Clustering techniques have helped researchers better understand antenna behavior under diverse environmental conditions, while predictive models are increasingly used to forecast satellite visibility and detect ionospheric disturbances. Collectively, these methods demonstrate how ML and DL are significantly enhancing GNSS accuracy and reliability in real-world operational settings.

In earthquake seismology, GNSS technology offers crucial advantages by accurately capturing ground movements from a geomatics perspective. Unlike traditional seismic sensors, which primarily record ground acceleration, GNSS uniquely measures both rapid and slow ground displacements, including subtle shifts that occur quietly over time [4,5,6]. This capability makes GNSS invaluable for monitoring the build-up of tectonic strain, studying ground movements during and after earthquakes, and examining how the Earth slowly adjusts following seismic events. Long-term GNSS monitoring networks, like Continuously Operating Reference Stations (CORS), provide detailed data that support both scientific research and effective hazard management strategies [4,5,6]. Additionally, when GNSS data are integrated with Geographic/Geospatial Information Systems (GIS), researchers can visualize seismic activity in great detail, significantly improving hazard assessments and emergency response planning [7].

GNSS has become a crucial part of modern Earthquake Early Warning (EEW) systems, especially because it helps overcome the limitations of traditional seismic instruments, like accelerometers, which can saturate or fail during intense shaking. Unlike these sensors, GNSS reliably measures both rapid ground movements and permanent displacements without becoming overwhelmed. Two main GNSS techniques have emerged as particularly useful: Precise Point Positioning (PPP) and Time-Differenced Carrier Phase (TDCP). PPP provides extremely accurate measurements by correcting satellite orbit and clock data, but it typically requires several minutes to produce stable results, making it less suitable for immediate earthquake alerts [13,14]. In contrast, TDCP can instantly determine ground velocities by comparing consecutive carrier-phase signals, offering almost immediate detection of seismic events [15]. Because of this rapid response capability, EEW systems such as ShakeAlert and Japan’s REGARD now utilize GNSS-derived TDCP velocities to swiftly estimate earthquake magnitudes, monitor rupture progress, and issue timely tsunami warnings [16,17].

Building on these established GNSS techniques, recent advancements in machine learning have further enhanced earthquake detection. For instance, Dittmann [11] developed an innovative machine learning framework specifically designed for GNSS-based seismic detection, using high-rate GNSS velocity data. To address the challenge of limited training data, a hybrid dataset combining real earthquake signals with simulated GNSS noise patterns was created. A random forest classifier was trained with this dataset using features such as peak velocities, low-frequency energy content, and waveform slope characteristics. This approach achieved better accuracy and reliability compared to traditional methods trained on smaller datasets [18]. Dittmann’s solution offers a scalable and robust option, particularly valuable in regions with sparse seismic monitoring infrastructure or limited observational data [11,18].

As machine learning continues to improve GNSS-based earthquake detection, understanding how these models make decisions has become increasingly important. Explainable Artificial Intelligence (XAI) techniques such as SHAP (SHapley Additive exPlanations) [19] and LIME (Local Interpretable Model-agnostic Explanations) [20] have emerged as valuable tools to clearly reveal how these advanced models arrive at their predictions. These XAI methods provide clear insights, showing exactly which features—such as satellite positions or signal quality—most strongly influence a model’s decisions. By improving transparency, XAI helps build trust in automated systems, which is crucial in real-time monitoring situations where clear explanations significantly impact decision-making. Although XAI in GNSS applications is still developing, early results suggest it has considerable potential to support more trustworthy and easily understood geospatial systems [19].

Building on these developments, researchers have begun applying Long Short-Term Memory (LSTM) networks to analyze GNSS data for earthquake monitoring. These specialized neural networks are designed to recognize patterns over time, making them well-suited for capturing temporal trends in ground movement [21]. From a practical geomatics standpoint, LSTM networks excel at detecting subtle yet critical temporal signals in GNSS data, such as minor ground movements before an earthquake, rapid shifts during seismic events, and slow movements afterward. Due to their built-in ability to remember important information over long sequences, LSTMs are especially suited for tracking gradual geological processes, like the slow build-up of tectonic strain or subtle ground shifts. Moreover, combining LSTM networks with XAI tools such as attention mechanisms [22] can further enhance our understanding of these processes. These combined methods make it clear to experts exactly which aspects of the data are most important for predictions, helping inform more accurate hazard assessments. Despite the promising results, comprehensive integration of LSTM with XAI techniques remains relatively new.

This study aims to build upon previous work such as Hohensinn’s [23], who employed GNSS-derived instantaneous velocities to detect potentially dangerous ground movements. While Hohensinn primarily used traditional statistical methods with GNSS data, our research significantly expands upon this foundation by introducing advanced machine learning methods, particularly LSTM networks, to better capture and model complex temporal patterns in GNSS signals. Additionally, by integrating -XAI method- SHAP based analysis, we seek to improve the clarity and interpretability of our results. Combining these advanced methods with pseudo-synthetic data approaches introduced by Dittmann [11], our goal is to provide a more accurate, reliable, and transparent system for GNSS-based earthquake detection, improving both scientific understanding and practical decision-making [23].

Motivated by the foundational work by Dittmann [11], our study aims to enhance the ability of GNSS-based systems to detect and classify earthquakes by integrating Long Short-Term Memory (LSTM) networks with Explainable Artificial Intelligence (XAI). While Dittmann successfully demonstrated the effectiveness of using random forest classifiers trained on high-rate GNSS observation-derived velocity time series data, we expand this approach by utilizing CNN and LSTM networks, which are particularly skilled at recognizing complex, evolving patterns in GNSS signals. By adding XAI techniques like SHAP [22], our method not only improves the accuracy of detecting seismic events but also makes it clear to users precisely why certain predictions were made. This integration offers a powerful tool for real-time earthquake monitoring, particularly in regions with limited seismic infrastructure. Although CNN, LSTM, and XAI methods have individually shown promising results, their combined application in Earthquake Early Warning (EEW) systems remains limited—an important gap our research addresses. 

Accordingly, this study contributes to GNSS-based earthquake monitoring by evaluating a hybrid CNN–LSTM architecture for seismic event detection from high-rate (5 Hz) GNSS velocity time series. In addition to frame-level metrics, we report event–station-level performance and examine operational decision choices. These include event-score aggregation (max, mean, and p90) and a vote-based fusion rule in which an event is declared detected when at least two of the three components (*H*0, *H*1, *UP*) predict a positive label. We further analyze operating points using a fixed reference threshold (τ = 0.5) and validation-set thresholds selected to maximize the F1-score (F1-optimal thresholds). This analysis illustrates the trade-off between sensitivity and false alarms under real GNSS noise conditions. We additionally examine validation-derived target-FPR thresholds as an ablation to further control false alarms. In this target-FPR analysis, max-based F1-optimal thresholds are applied for the horizontal components, whereas a mean-based target-FPR threshold is applied for the vertical component. Moreover, we use MMD- and SHAP-based analyses to describe the distributional and attributional differences between the training and test sets. We report these results as supportive diagnostics alongside the detection metrics. Finally, to enable a direct comparison, we evaluate the hybrid model together with an RFM baseline and the CNN-only and LSTM-only variants using the same data splits, preprocessing, and evaluation metrics.

In summary, the main contributions of this study can be summarized as follows:

1. We evaluate an end-to-end hybrid CNN–LSTM model for seismic detection from raw 5 Hz GNSS velocity time series on an independent real GNSS test setting that includes realistic ambient (non-event) noise. We contextualize the results with concise baseline comparisons (RFM, CNN-only, and LSTM-only).
2. We report performance at both frame level and event–station level, and we define an event–station scoring procedure that aggregates frame-level predictions within each event–station pair.
3. We examine operational decision choices that govern event–station behavior, including multi-component fusion (with vote-based 2-of-3 as the primary decision rule) and alternative event-score aggregation strategies (e.g., max/mean/p90). We also report results at two operating points: a fixed reference threshold (τ = 0.5) and a validation-set threshold selected to maximize the F1-score (F1-optimal operating point).
4. We complement detection results with distribution and attribution diagnostics (MMD and SHAP) to characterize train–test differences and support interpretation of generalization behavior under realistic noise conditions.

## 2. Materials and Methods

### 2.1. Data Preparation and Preprocessing

In this study, high-rate (5 Hz) GNSS velocity time series data were used for seismic event detection. The main training resource was the pseudo-synthetic GNSS velocity feature set generated by Dittmann et al. [11]. The feature set is a combination of real seismic waveforms and realistically modeled GNSS noise (Mw ≥ 5.0). Using this dataset in the training of our model allowed us to broaden the range of training conditions where real events are relatively limited. As the test resource, real GNSS velocity time series generated by Dittmann et al. were also used [18].

In their original study, visually labeled real GNSS velocity time series were used within Random Forest Machine Learning analysis, while the corresponding visual annotations were not distributed with the raw data [18]. In a subsequent study, the same velocity time series data and their existing visual annotations were released in the form of a labeled real-data feature set together with the pseudo-synthetic feature sets and pseudo-synthetic time series data.

Since our CNN-LSTM model operates directly on raw GNSS velocity time series, both labeled synthetic time series and real-event GNSS velocity time series were required. To ensure consistency with the preprocessing strategies of the real feature set, real velocity time series were segmented using the same index-based windowing approach (30 s windows with a 10 s sliding step at 5 Hz). Velocity time-series windows were paired one-to-one with rows of the corresponding real-data feature set based on window index. Thereafter, associated frame-level labels were assigned accordingly. Samples with inconsistent window counts were excluded, if present. Because labels were defined at the frame level in the original feature set, no special handling was applied for P-wave on-sets falling near window boundaries.

Furthermore, following the recommended use of synthetic data set with additional ambient conditions [18], regarding synthetic dataset usage, we gathered ambient data from EarthScope GNSS RINEX files recorded at a 5 Hz sampling rate. Specifically, ambient data from one-hour intervals preceding the real seismic events were used (956 for training; 1265 for testing). Ambient data were selected from the same earthquake–station pairs as in the real-event dataset. Ambient data were segmented into 30 s windows with a 10 s sliding interval. Training ambient data consist of 5 min (from 47 stations) and 10 min long (from 909 stations) data. Test ambient data duration was also 10 min (from 1265 stations). (Differences in the number of available pre-event ambient sites arise from differences in retrieval times.) This created a comprehensive non-event labeled ambient dataset for training, enhancing our model’s capability to distinguish seismic events from ambient conditions.

Each GNSS velocity time-series segment represented above is associated with a single station and a given earthquake. Performance is evaluated at the frame level and at the station level, where sliding-window predictions are combined within each station independently, without aggregation across stations (Table 1).

The synthetic train/validation partitions are defined at the station–event waveform level prior to sliding-window extraction; therefore, all overlapping windows from a given waveform remain within the same split, preventing leakage under the 10 s stride.

In the real test set, after excluding padded values, the frame-level class distributions (0/1) were as follows: *H*0: 509,541 non-events vs. 25,554 events; *H*1: 511,527 non-events vs. 23,568 events; and *UP*: 531,441 non-events vs. 3654 events. At the event–station level (N = 2966 groups), the positive/negative counts are *H*0 1118/1848; *H*1 1071/1895; and *UP* 189/2777.

To ensure uniformity and facilitate meaningful comparisons across various seismic events, global z-score normalization, a technique well-established in previous GNSS research, was applied [11,24].

The normalization parameters (*μ*_*train*, *σ*_*train*) were computed using the training split (synthetic train augmented with ambient non-event data) only, and then the same parameters were applied to the validation and real test splits to avoid information leakage.

The channel-wise global Z-score normalization technique was used to standardize each GNSS velocity time series channel independently. By computing the mean (μ_tain) and standard deviation (σ_train) of each channel (component) from the training split, Z-score normalization transforms each data point x according to

(1) $$Z=\frac{x-\mu \_train}{\sigma \_train}$$

where

- x: The original GNSS velocity measurement.
- *μ*_*train*: The mean of the training split for the corresponding GNSS velocity channel.
- *σ*_*train*: The standard deviation of the training split for the corresponding GNSS velocity channel.
- *Z*: The resulting normalized value with zero mean and unit variance.

This process standardizes the dataset inputs, ensuring consistent scaling across channels, facilitating effective training and improving numerical stability for neural network models [11].

### 2.2. Hybrid CNN-LSTM Model Architecture

In this study, we employed a one-dimensional convolutional neural network (1D-CNN) architecture to analyze multivariate GNSS velocity time-series data. The three components (*H*0, *H*1, and *UP*) are processed jointly to identify local patterns and abrupt changes. To enlarge the effective receptive field without increasing the parameter count, two consecutive 1D convolutional layers were utilized. This design introduced the necessary non-linearity for more expressive feature learning. The first layer focuses on extracting short-scale signal motifs, including abrupt onsets or brief oscillations, whereas the second layer integrates these localized patterns into higher-level features. In other words, the model learns the localized features within each 30 s window for subsequent temporal modeling. In addition, interleaved max-pooling operations were employed to enhance the network’s stability and produce compact feature maps. This design helps suppress noise effects in GNSS data. To accommodate variable-length window sequences, inputs were padded to a fixed length and a Boolean mask was derived to mark valid windows. This mask was used to restrict feature extraction to valid windows. The same mask was propagated to the subsequent recurrent layers so that padded windows did not influence temporal modeling. The feature extraction block consists of two consecutive Conv1D layers (kernel size 5, ReLU activation, same padding) interleaved with max-pooling layers (pool sizes 3 and 2). The resulting feature maps are then flattened and used as inputs for the subsequent recurrent block.

We placed the LSTM block after the CNN-based feature extractor to learn the temporal dynamics of the extracted localized features across window sequences. We used two stacked bidirectional LSTM (BiLSTM) layers to capture both short- and long-term temporal dependencies within the GNSS velocity time series channels [21]. The first BiLSTM layer uses N units and returns sequences, preserving the temporal structure of the extracted feature maps. The second BiLSTM layer uses a reduced width (with a minimum of 32 units) and also returns sequences. Dropout regularization was applied between the layers with rates p and 0.7p to improve generalization and prevent overfitting.

Separate predictions are produced for each GNSS velocity component (*H*0, *H*1, and *UP*). The recurrent features are then mapped to probabilities through a small dense classification stage, followed by a time-distributed sigmoid output for each component. In this way, seismic event detection is formulated as a multi-output binary classification task.

### 2.3. Training Procedure

The final hybrid CNN–LSTM model was trained on 30 s segments of GNSS velocity time series sampled at 5 Hz for three channels (*H*0, *H*1, and *UP*). Each channel’s input sequence was standardized using global Z-score normalization computed on the training split and then applied to the validation and test splits to ensure consistent scaling across partitions. During training, padded windows were assigned zero sample weights so they did not contribute to the loss. In addition, masking ensured that padded windows did not influence the recurrent temporal modeling.

For optimization, we used the Adam optimizer with a learning rate of 9.23 × 10^−4^ selected by Optuna. The model was trained for up to 100 epochs, using early stopping and learning-rate scheduling. Early stopping monitored the validation macro PR-AUC and restored the best weights. The learning rate was reduced on plateaus of the same metric.

To handle channel imbalance, we used a weighted binary cross-entropy objective. Model selection was based on the highest validation macro PR-AUC. Per-channel best-F1 thresholds were determined on the validation set and used only for performance reporting in the evaluation stage.

(2) $$L_{train\;}=\sum_{c\in \left\{H0,H1,UP\right\}}\omega_{c}\cdot BCE(y_{c,\;}{\hat{y}}_{c})$$

where $\omega_{H0}=0.3,\;\omega_{H1}=0.3,and\;\omega_{UP}=0.4$.

Because early P-wave motion is most effectively captured in the vertical (*UP*) component, a slightly higher loss weight was assigned to *UP* [25,26]. Model selection and hyperparameter optimization were conducted independently of this loss weighting, using the validation macro PR-AUC as the sole selection criterion computed as the unweighted mean of per-channel PR-AUC values. This separation ensures that channel weighting affects only the optimization process during training, while model selection remains agnostic to individual GNSS components.

#### 2.3.1. Hyperparameter Optimization

We performed hyperparameter optimization with the Optuna framework using a fixed training/validation split [27]. The optimization objective was defined as the validation macro PR-AUC, which served as the sole criterion for comparing different configurations.

The search space covered both architectural and training-related parameters. Specifically, the number of convolutional filters and LSTM units, reduction factors applied to the CNN and LSTM blocks, learning rate, dropout rate, and batch size were explored. The corresponding search space was defined as follows: CNN filters ∈{64,128,256}, CNN reduction factor ∈{1,2}, LSTM units ∈{64,128,256}, LSTM reduction factor ∈{1,2}, learning rate $\in [1\times 10^{-5},1\times 10^{-3}]$ sampled on a logarithmic scale, dropout rate ∈[0.1,0.5], and batch size ∈{32,64,128}.

A total of 25 Optuna trials were conducted. Each trial was trained for up to 20 epochs with early stopping based on the validation macro PR-AUC (patience = 10). In addition, a median pruning strategy was used to terminate underperforming trials during hyperparameter optimization.

All remaining architectural components and training settings were kept fixed during the optimization process.

#### 2.3.2. Evaluation Metrics and Event Prediction

Model performance was evaluated primarily using precision–recall-based metrics for each channel, reflecting the class imbalance inherent in seismic detection. As the main criterion for performance monitoring and model selection, the per-channel PR-AUC and the derived macro PR-AUC (unweighted mean across channels) were computed [28,29]. In addition, precision, recall, F1-score, and average precision (AP) were calculated for providing complementary insight into detection quality. Confusion matrices and precision–recall curves were generated for validation reporting and visual assessment of model discrimination performance. For metrics requiring binary decisions, results were computed on the validation set using both a fixed threshold of 0.5 and per-channel best-F1 thresholds; these thresholds were used only for reporting and comparison, and were not used for model selection.

### 2.4. SHAP-Based Attribution Analysis

To provide a supportive diagnostic of the final hybrid CNN–LSTM model’s behavior, we integrated a SHAP-based attribution analysis into our framework. This analysis complements the distributional shift determination by providing an attribution-based perspective on model behavior under changed test conditions. SHAP values were computed using the GradientExplainer implementation for the gradient-based hybrid CNN–LSTM model [19]. The explainer was initialized with a compact background set randomly sampled from the validation data (128 windows). SHAP values were then computed for a validation subset (256 windows) and for a 1000-window subset of the test set to compare attribution magnitudes between validation and test conditions. For each GNSS channel (*H*0, *H*1, *UP*), channel-wise mean absolute SHAP values were reported as an aggregate diagnostic. This aggregate summary provides a compact, channel-level diagnostic for comparing attribution magnitudes between validation and test conditions.

## 3. Results and Discussion

The proposed hybrid CNN–LSTM model was developed and trained using the synthetic time-series dataset constructed by Dittmann et al. [11]. The dataset integrates real seismic ground motion recordings with statistically modeled GNSS noise and was designed to simulate strong-motion observations under realistic noise conditions. Furthermore, real GNSS velocity time series data from 77 seismic events (2005–2021)—previously published by Dittmann for training a Random Forest Model (RFM)—were used to test our hybrid model [18].

Additionally, we obtained real ambient data from EarthScope as raw GNSS data (5 Hz) for training and testing. These data were extracted from one-hour intervals preceding the earthquake occurrence times at the same event–station pairs in the real dataset. To further increase the size and diversity of the training ambient dataset, we selected eight additional earthquakes occurring between 2021 and 2025. These additional earthquakes were selected from regions aligned with the regions represented in the real dataset. Following the site-selection strategy used for the published real-event dataset, we included all available GNSS sites within ~70 km of each selected earthquake epicenter, totaling 47 sites. Almost 95% of the gathered site data were 10 min long, and the remaining were 5 min long.

The raw 5 Hz GNSS observations were converted into velocity time series using the SNIVEL tool. Thereafter, we turned the data into 30 s windows (with a 10 s sliding). The real ambient (non-event) data were labeled as zero (0). On the other hand, we first windowed the real (event) velocity time series [18]. Then, we aligned it with the real feature set [11] that was produced from the visually labeled real velocity time series.

In summary, both non-event and event data were prepared as windowed raw time series with binary labels (0 for non-events, 1 for events), forming the complete input for CNN–LSTM model training and testing.

### 3.1. Model Training and Validation Performance

The final hybrid CNN–LSTM model was trained using the synthetic time series. In addition, the training dataset was enriched with the real ambient data (used as non-seismic background).

The training process was designed to run for up to 100 epochs, with early stopping and learning-rate scheduling. The training and validation curves for both accuracy and loss plateaued around epoch ≈ 25–30, indicating efficient optimization and appropriate regularization. The final model was selected based on the highest macro PR-AUC score of the validation set.

The model demonstrated smooth learning behavior throughout training. On the validation data, the macro PR-AUC reached 0.948 across all channels (*H*0, *H*1, *UP*). These values are slightly lower than the corresponding training metrics, and showed consistent performance for each epoch. The channel-wise metrics are presented in Table 2.

**Figure 1:**
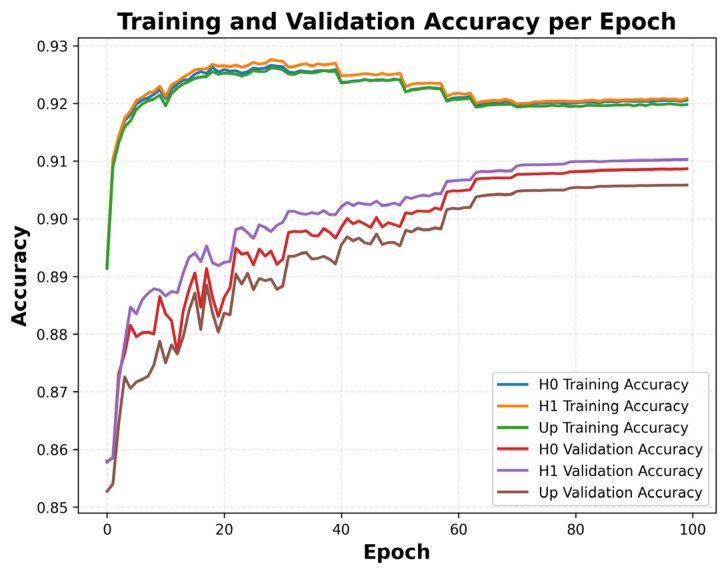
Frame-level training and validation accuracy per epoch for the hybrid CNN–LSTM model (*H*0, *H*1, and *UP* components) using the synthetic hybrid dataset.

As shown in Figure 1, the close alignment between training and validation metrics suggests that the model generalized well to unseen data, with no significant signs of overfitting. These results indicate that the hybrid CNN–LSTM architecture achieved both stable optimization and reliable generalization performance in the context of GNSS velocity time-series-based seismic event detection.

**Figure 2:**
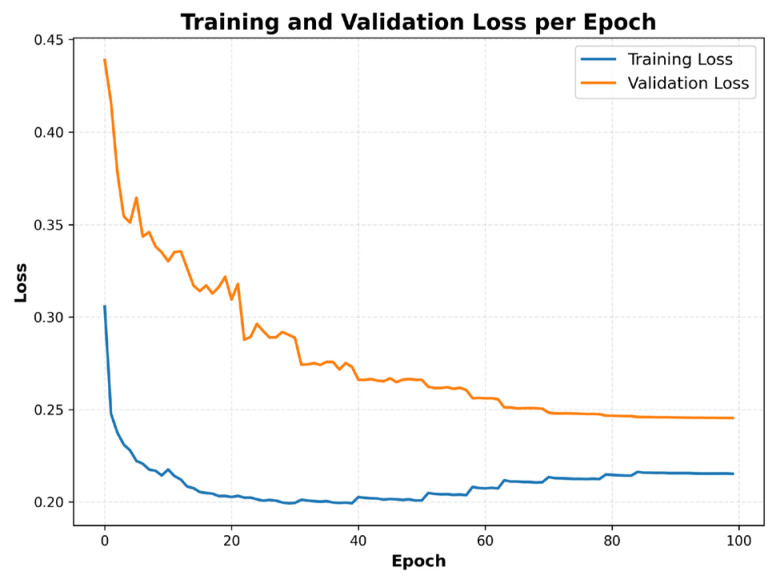
Training and validation loss per epoch: binary cross-entropy loss curves illustrating steady convergence and close alignment between training and validation losses.

The binary cross-entropy loss curves demonstrated a stable learning trend throughout training (Figure 2). Both training and validation losses decreased steadily and plateaued after around epoch ≈ 5. The downward trajectory of the training loss indicated effective learning. Meanwhile, fluctuations in the validation loss reflected the model’s response to unseen data.

**Figure 3:**
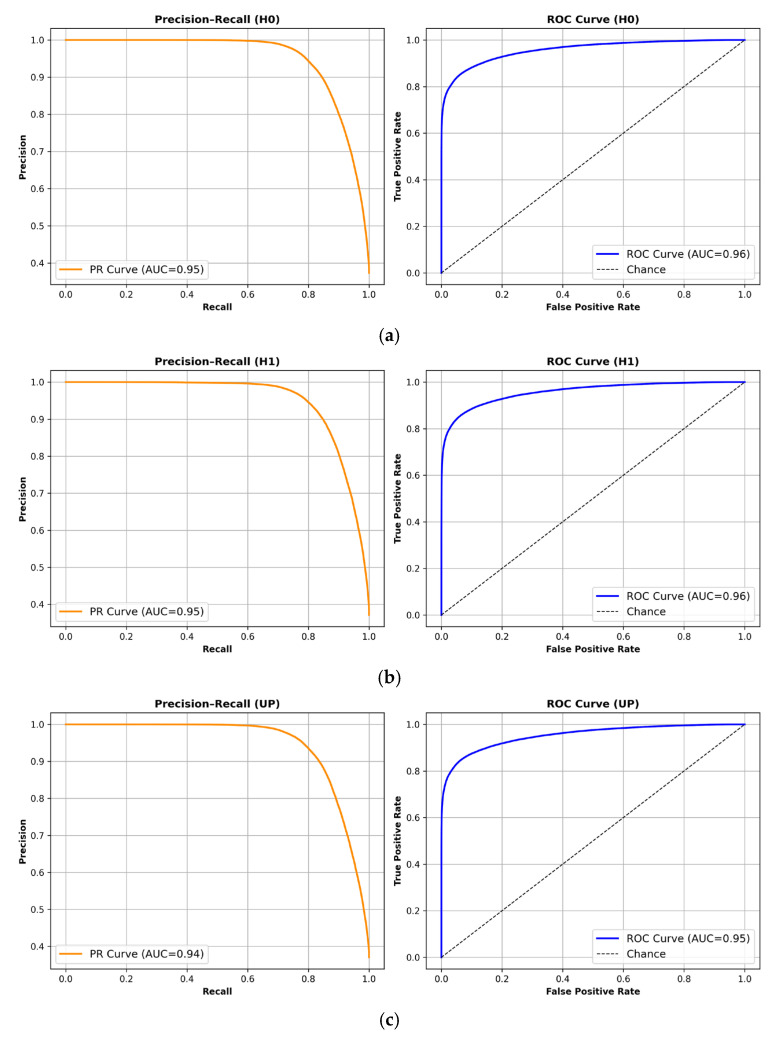
Frame-level precision–recall (left) and ROC (right) curves for *H*0, *H*1, and *UP* components using the synthetic hybrid dataset. (**a**) *H*0 channel, (**b**) *H*1 channel, (**c**) *UP* channel.

Precision–recall and ROC curves for the three GNSS velocity components (*H*0, *H*1, *UP*) are shown in Figure 3.

**Figure 4:**
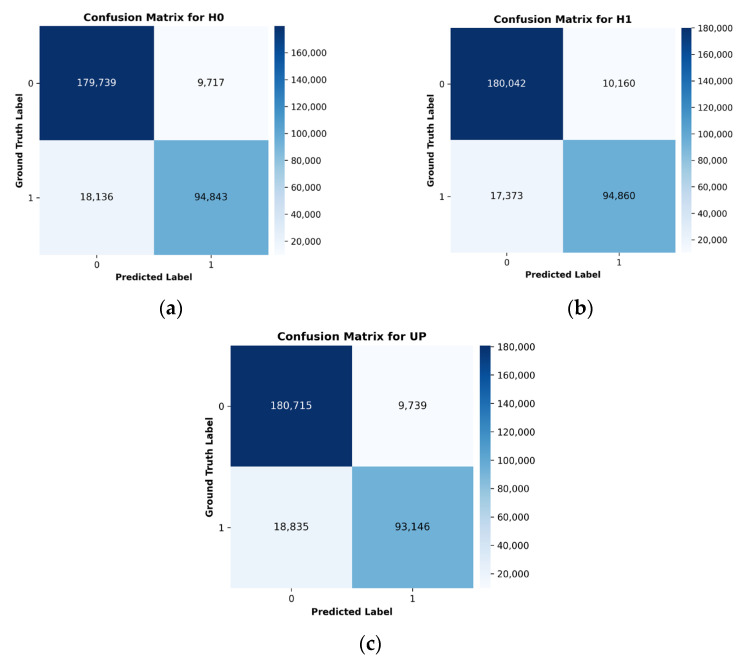
Frame-level validation confusion matrices for the *H*0, *H*1, and *UP* components using the synthetic hybrid dataset. (**a**) *H*0 channel, (**b**) *H*1 channel, (**c**) *UP* channel.

As shown in Figure 4, the confusion matrices present the validation performance of the developed CNN-LSTM model across three GNSS velocity channels (*H*0, *H*1, and *UP*). Each matrix provides a clear visualization of correct and incorrect predictions made by the model.

Summary of Channel Performance:

- Channel *H*0: Lowest false positives.
  ▪ True Negatives (TN): 179,739 instances correctly identified as non-events.
  ▪ False Positives (FP): 9717 non-event instances incorrectly classified as events.
  ▪ False Negatives (FN): 18,136 event instances incorrectly classified as non-events.
  ▪ True Positives (TP): 94,843 event instances correctly classified.
- Channel *H*1: Most balanced performance with the lowest false negatives.
  ▪ True Negatives (TN): 180,042 instances correctly identified as non-events.
  ▪ False Positives (FP): 10,160 non-event instances incorrectly classified as events.
  ▪ False Negatives (FN): 17,373 event instances incorrectly classified as non-events.
  ▪ True Positives (TP): 94,860 event instances correctly classified.
- Channel *UP*: Comparable performance with slightly higher false negatives.
  ▪ True Negatives (TN): 180,715 instances correctly identified as non-events.
  ▪ False Positives (FP): 9739 non-event instances incorrectly classified as events.
  ▪ False Negatives (FN): 18,835 event instances incorrectly classified as non-events.
  ▪ True Positives (TP): 93,146 event instances correctly classified.

Overall, these confusion matrices demonstrate the model’s classification performance for all channels. The *UP* channel exhibits slightly lower false positive rates, while *H*0 and *H*1 show balanced detection behavior.

In addition to macro PR-AUC, we computed composite scores and auxiliary metrics—such as average precision (AP), false-positive rate (FPR), and detection latency—to provide a more comprehensive view of model performance and diagnostic analysis only. Therefore, these secondary metrics did not influence model selection process.

### 3.2. Hyperparameter Optimization via Optuna

For hyperparameter tuning, using Optuna, 25 candidate configurations were evaluated. The best-performing configuration (Trial 11) achieved a validation macro PR-AUC of 0.9501.

The corresponding channel-wise validation PR-AUC values were 0.951 for H0, 0.951 for H1, and 0.948 for UP. The optimal hyperparameter configuration identified by Optuna is summarized in Table 3. The channel-wise validation PR-AUC results are presented in Table 4. This configuration was subsequently adopted for the final model training.

### 3.3. Final Model Testing

The final evaluation was conducted on the independent real GNSS test dataset using both event–station-based and frame-level (window-level) evaluation schemes.

For both evaluation levels, results are reported under two operating points. The first is a fixed global threshold (τ = 0.5), uniformly applied to all channels. The second consists of channel-wise validation-optimized thresholds. These thresholds are selected by maximizing the F1-score on the validation data.

Additionally, the validation performance-based threshold selection procedures differ between the two evaluation schemes. For event–station-based evaluation, thresholds are obtained by re-optimizing the F1-score on event-level scores. These scores are computed by aggregating frame-level predictions within each event–station pair. The resulting event-level scores are then combined across channels using different fusion strategies. We evaluated a vote-based rule (2-of-3 channels), an any-channel (OR) rule, and a weighted fusion scheme. Since the alternative fusion strategies (any-channel and weighted) yielded nearly identical overall trends, we report the vote-based (2-of-3) rule in the main table for clarity. The remaining fusion variants are provided as ablation results. Under the fixed threshold (τ = 0.5), the any-channel, vote (2-of-3), and weighted fusion rules yield nearly identical performance (differences at the third decimal place), so we report the vote-based rule in the main table for clarity. The corresponding event–station-level channel performance metrics based on max-aggregated event scores and vote-based rule are summarized in Table 5. Table 6 then presents the event–station-level fused performance of the proposed CNN–LSTM model under the vote-based (2-of-3) fusion rule. 

At the two operating points (τ = 0.5 and val-bestF1), the corresponding FPR values (0.29 and 0.42) are computed from the confusion matrices of the fused (vote 2-of-3) decisions. For completeness, additional fusion variants (any-channel and weighted) are provided as ablation results in Table S1, and alternative aggregation modes (mean and p90) are summarized in Table S2. Additionally, the confusion-matrix counts (TN/FP/FN/TP) used to compute the operating-point metrics (including FPR) for the fusion variants are provided in Table S1 for transparency.

As an ablation on operating-point selection, we evaluated a channel-dependent hybrid thresholding strategy. The horizontal components (*H*0, *H*1) used max aggregation with validation-optimized best-F1 thresholds. The vertical component (*UP*) used mean aggregation with a validation-calibrated target-FPR threshold (0.05). At the event–station level, these setting yields TP/FP/TN/FN of 1118/856/992/0 for *H*0 and 1071/903/992/0 for *H*1, corresponding to precision of 0.566 and 0.543 with recall of 1.000 for both channels. For *UP*, TP/FP/TN/FN is 170/694/2083/19 (precision 0.197, recall 0.899), indicating that the vertical component remains the dominant source of missed detections under this operating-point constraint.

Following the event–station-based analysis, we additionally examine the frame-level performance to characterize the model’s discrimination behavior prior to event aggregation (Table 7).

Overall, the hybrid model exhibits reduced detection performance under real GNSS noise compared to synthetic training conditions.

### 3.4. Data Distribution and Model Behavior Analysis

Accordingly, in this section we investigate distributional differences between the training and test datasets. In addition, we examine their potential implications for the model’s decision behavior.

#### 3.4.1. Distributional Shift Quantification

**Figure 5:**
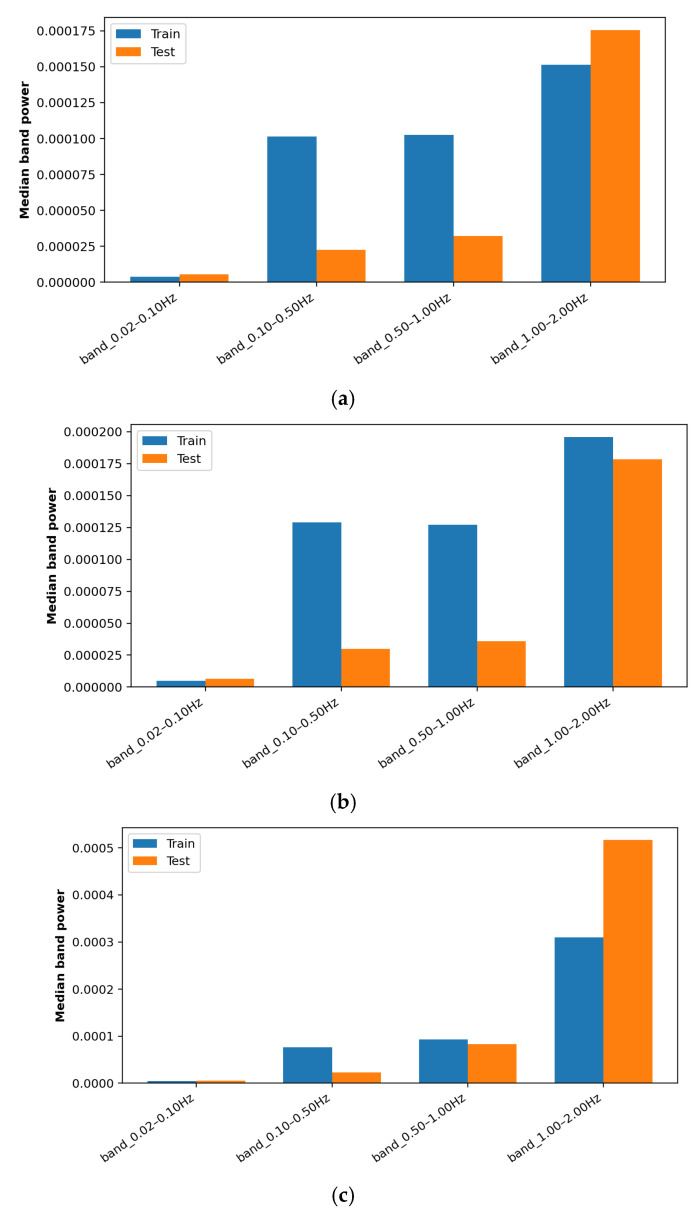
Median PSD band power for the combined train and validation set (labeled as “Train”) and test set across four frequency bands. (**a**) Median PSD band power of *H*0, (**b**) median PSD band power of *H*1, (**c**) median PSD band power of *UP*.

To quantify potential distributional differences between the train set (including both train and validation) and test set conditions, we extracted channel-wise descriptive and spectral features. The descriptive statistics include measures of central tendency, dispersion, and quantile-based range calculations. For spectral features, Welch-based Power Spectral Density (PSD) estimates were computed over predefined signal (0.10–1.50 Hz) and noise (1.50–2.30 Hz) frequency bands, considering frequency ranges reported in previous studies [4,11,24]. The median band-power comparisons between the trainset (train and validation; labeled “Train” in Figure 5) and test sets further illustrate these spectral differences (Figure 5). A simple signal-to-noise ratio (SNR) estimate was also included to reflect noise conditions. Since all feature vectors were already globally normalized during preprocessing, the train and test set distributions were compared using an RBF-kernel Maximum Mean Discrepancy (MMD) metric. The resulting channel-wise MMD estimates (with 95% confidence intervals) are summarized in Table 8.

The observed MMD values indicate a statistically significant shift between the train and test distributions. For the horizontal *H*0 and *H*1 channels, MMD values reached 0.105 and 0.094, respectively, and both were supported by narrow 95% confidence intervals (*H*0: 0.100–0.111; *H*1: 0.090–0.099). The *UP* channel exhibited a relatively smaller shift, MMD score of 0.066 and 95% confidence interval of 0.062–0.071. These results indicate differences in the spectral and noise characteristics between the training and test datasets. The differences are aligned with the observed performance gap on real GNSS data.

Moreover, the following analysis examines how this distributional shift alters the model’s decisions.

#### 3.4.2. SHAP-Based Model Behavior Analysis

To complement the distributional comparison provided by MMD, we further used SHAP to examine whether the model’s attribution magnitudes change under the shifted test condition. While MMD compares the training and test distributions, the SHAP analysis contrasts validation and test conditions to quantify how attribution magnitudes evolve when moving from validation to test. The analysis is summarized at an aggregate level across the output channels, without focusing on individual features. This perspective allows for a comparison of attribution behavior under different data distributions. In this context, SHAP results are used as a descriptive tool to support the examination of potential domain shift effects on the model’s behavior.

To further examine the model’s decision behavior, channel-wise SHAP values were computed for the validation and test tensors. A GradientExplainer was applied, and mean absolute SHAP values were computed for each channel. Compared to the validation set, the test set exhibited higher mean absolute SHAP values.

We conducted the analysis with a 1000-window subset sampled from the 107,019 test windows. For the validation and test H0 channels, the mean absolute SHAP values increased from 0.85 $\times \;10^{-3}$ to 2.59 $\times \;10^{-3}$, while the *H*1 channel increased from 1.07 $\times \;10^{-3}$ to 2.82 $\times \;10^{-3}$, respectively. The *UP* channel of the validation and test set exhibited the largest shift, increasing from 1.17 $\times \;10^{-3}$ to 3.08 $\times \;10^{-3}$. These results demonstrate a consistent rise in attributions across all channels, with the *UP* channel being the most affected. The inflation ratios range from approximately 2.64× to 3.04× when using the 1000-window test subset (Table 9).

In summary, the distributional shift identified by MMD analysis is further supported by the SHAP results. This convergence of findings indicates that the reduction in detection performance results from a mismatch in data characteristics. The observed model behavior under real GNSS noise conditions is consistent with the identified distributional differences. This outcome aligns with observations reported in previous studies [4,11,24].

### 3.5. Final Model Performance and Limitations

The final evaluation on the independent real GNSS test set showed a clear reduction in detection performance relative to the validation regime. On the validation set, the macro PR-AUC reached 0.948 across the three channels (*H*0: 0.950, *H*1: 0.950, *UP*: 0.945). On the real test set, performance depended on the operating point and the evaluation level.

For the primary evaluation, results are reported at the event–station level. In this setting, frame-level predictions are aggregated into a single score per event–station pair (per channel) and then fused across channels. In the main analysis, we use max aggregation and the vote-based 2-of-3 fusion rule.

To make the false-alarm cost explicit, we report two operating points on the real test set. The first is a fixed reference threshold (τ = 0.5). The second uses validation-calibrated thresholds (val-bestF1) selected on the validation set and then applied unchanged to the test set, i.e., without any test-time tuning. Under 2-of-3 voting with max aggregation, PR-AUC scores were 0.834 at τ = 0.5 and 0.815 at val-bestF1 (Table 6). Using the val-bestF1 thresholds, the fused decision achieved recall = 1.000, while the corresponding false-positive rate under real ambient noise increased relative to τ = 0.5 (Table 6). These results illustrate a trade-off between missed detections and false alarms under real ambient GNSS noise. Therefore, we report τ = 0.5 with vote-based (2-of-3) fusion as the primary operating point on the real test set, and include val-bestF1 as a validation-derived reference to contextualize the recall–FPR trade-off.

Channel-wise event–station results indicate different performance levels across components under real noise. For τ = 0.5, PR-AUC was 0.817 for *H*0, 0.796 for *H*1, and 0.484 for *UP* (Table 5). The corresponding event–station-level F1-scores were 0.765 (*H*0), 0.750 (*H*1), and 0.204 (*UP*) (Table 5). Overall, performance on the real test set is lower than on validation, with the largest degradation observed for the vertical (*UP*) component.

The pronounced degradation observed on the *UP* (vertical) component is consistent with prior high-rate GNSS studies. These studies report that the vertical component typically exhibits higher ambient noise and reduced stability relative to the horizontal components [11,18]. This is consistent with the generally poorer vertical precision observed in GNSS positioning/velocity solutions [24]. This can broaden the *UP* model-score distribution and reduce practical separability relative to *H*0/*H*1. For EEW applications, this limitation can affect reliability. Under a given threshold setting, treating UP identically to *H*0/*H*1 can alter the balance between missed detections and false alarms. Operationally, lowering thresholds to recover sensitivity may increase false alarms, whereas raising thresholds to control false alarms may increase missed detections. Therefore, channel-dependent decision logic can be calibrated to the network’s false-alarm tolerance through channel-specific thresholding and fusion-rule design (e.g., vote-based or weighted fusion). If site-specific validation indicates that the *UP* channel is persistently noise-dominated, an *H*0/*H*1-only triggering configuration can be considered as a deployment-dependent option. In this configuration, *UP* is excluded from the triggering rule.

We also report ablation results to contextualize the evaluation choices. Alternative fusion rules (any-channel and weighted) and alternative aggregation modes (mean and p90) were examined (Tables S1 and S2). Across these ablations, the relative interpretation is mainly influenced by the operating-point choice (τ = 0.5 versus val-bestF1). We additionally explored a channel-dependent thresholding variant to control false alarms. In this setting, validation-derived best-F1 thresholds applied for the horizontal components (*H*0 and *H*1) using max aggregation. For the vertical component (*UP*), a validation-derived target-FPR threshold was applied using mean aggregation. This hybrid thresholding strategy was implemented to reflect the component-dependent score behavior observed in our analysis. These variants are reported as ablation results and are not used for the primary operating point.

The model was also evaluated at the frame level under real GNSS noise conditions using the same protocol as in training and validation. Frame-level performance remained low, indicating limited separability at this finer temporal resolution. Accordingly, event–station aggregation was retained as the primary reporting level to better reflect event–station behavior under realistic noise.

In addition to the main model evaluation, the proposed CNN–LSTM hybrid model was compared with baseline approaches on the same datasets, including a Random Forest Model (RFM), a CNN-only variant, and an LSTM-only variant. The RFM baseline follows the publicly available implementation provided by Dittmann et al. [11] and uses a combined time–frequency feature set, where summary statistics and spectral features are concatenated across components. In contrast, the CNN-only, LSTM-only, and hybrid CNN–LSTM models are trained end-to-end on raw 5 Hz GNSS velocity time series.

A quantitative comparison of the proposed CNN–LSTM hybrid model and the baseline approaches is presented in Table 10 under the event–station-level evaluation setting. Results are reported at the fixed operating point (τ = 0.5) for the deep learning models, while the RFM baseline is included using the operating point and evaluation procedure of the original implementation [11].

At the selected operating points, the models exhibit different precision–recall trade-offs. The hybrid model tends to favor recall compared to the CNN-only and LSTM-only variants, leading to higher sensitivity but moderately lower precision than the LSTM-only model and a slightly lower F1-score. The LSTM-only model achieves the highest F1-score among the deep learning variants in this comparison. The RFM baseline shows high recall with lower precision, resulting in a lower F1-score despite a high PR-AUC. Overall, these results reflect differences in operating characteristics rather than clear performance dominance across models.

These results demonstrate that a purely time-series-driven CNN-LSTM training scheme is sensitive to distributional shifts under GNSS noise conditions, in alignment with previous studies. Consequently, future training strategies should include spectral and noise-aware features to reduce this sensitivity. In this regard, the present contribution represents an initial methodological step toward hybrid CNN-LSTM-based P-wave detection with high-rate GNSS velocity time series.

## 4. Conclusions and Future Directions

The hybrid CNN–LSTM model developed in this study investigates high-rate (5 Hz) GNSS velocity time series for seismic event detection. The model was trained and validated using a hybrid dataset that combines pseudo-synthetic seismic signals with real ambient GNSS noise. It was subsequently evaluated on an independent real GNSS test set containing real events and corresponding pre-event ambient segments recorded at the same stations. Validation performance indicated higher discrimination under the training/validation regime. However, the independent real test set did not reproduce the same level of performance, suggesting a distributional mismatch between pseudo-synthetic training conditions and real-world GNSS noise.

To ensure an interpretable evaluation under realistic noise, both frame-level and event–station-level evaluations were conducted. Because frame-level performance was lower on the real GNSS test set, event–station-level results were selected as the primary reporting level. In the main analysis, event–station scores are computed using max aggregation and fused using the vote-based (2-of-3) rule; alternative aggregation modes are reported as ablations. Additionally, two operating points were examined on the real test set: a fixed reference threshold (τ = 0.5) and a validation-derived operating point (val-bestF1). Using val-bestF1, the fused decision achieved higher recall and an increased false-positive rate on the real test set. Using τ = 0.5, the fused decision achieved lower recall and a lower false-positive rate on the real test set. Given the increased false-positive rates observed under val-bestF1 on the real test set, τ = 0.5 was retained as the primary reporting operating point to better reflect false-alarm cost, while val-bestF1 was used to illustrate the recall–FPR trade-off.

To further investigate the performance drop, we conducted MMD- and SHAP-based analyses. These analyses revealed a measurable distributional shift and a corresponding change in the model’s decision patterns.

Future work should focus on improving robustness to the observed train–test distribution shift under real GNSS noise. This could be pursued by complementing the current time-domain representation with PSD/PPSD-type descriptors and/or evaluating attention-based (transformer-style) sequence models. Moreover, refining the existing validation-based thresholding to satisfy predefined false-alarm tolerances (e.g., an FPR limit) while accounting for detection latency could improve the suitability of the model for EEW-oriented use.

## Figures and Tables

**Table 1:** Dataset summary.

Dataset Split	Unit of Analysis	Count	Description
Synthetic—Train	Station–event GNSS waveforms	11,895	Each waveform corresponds to one station recording of a synthetic earthquake
Synthetic—Validation	Station–event GNSS waveforms	2851	Used for hyperparameter selection
Real Test	Station–event GNSS waveforms	2966	Independent real GNSS Site recordings
Real Test	Unique earthquakes	77	Unique event IDs
Real Test	Sliding windows (30 s)	107,019	Extracted with 10 s stride
Train	Valid time steps (frame-level)	1,350,074	Excluding padded values
Validation	Valid time steps (frame-level)	302,435	Excluding padded values
Test (frame-level)	Valid time steps (frame-level)	535,095	Excluding padded values

**Table 2:** Channel-wise validation PR-AUC scores of the final hybrid CNN–LSTM model.

Metric	*H*0	*H*1	*UP*	Macro PR-AUC
PR-AUC (validation)	0.950	0.950	0.945	0.9481

**Table 3:** Hyperparameter search space and optimal configuration selected by Optuna.

Hyperparameter	Search Space	Optimal Value
CNN filters	{64, 128, 256}	128
CNN reduction factor	{1, 2}	1
LSTM units	{64, 128, 256}	256
LSTM Reduction Factor	{1, 2}	1
Learning rate	[1 × 10^−5^, 1 × 10^−3^] (log-uniform)	$9.24\;\times \;10^{-4}$
Dropout rate	[0.1, 0.5]	0.26
Batch size	{32, 64, 128}	128
Optimization objective	–	Validation macro PR-AUC

**Table 4:** Channel-wise validation PR-AUC scores of the best Optuna configuration for the hybrid CNN–LSTM model.

Metric	*H*0	*H*1	*UP*	Macro PR-AUC
PR-AUC (validation)	0.951	0.951	0.948	0.9501

**Table 5:** Event–station-level channel performance metrics for both val-bestF1 and τ = 0.5 operating points.

Channels	Decision Threshold	Precision	F1	Recall	PR-AUC
*H*0 (North)	0.1007	0.566	0.723	1.000	0.817
*H*1 (East)	0.1031	0.543	0.703	1.000	0.796
*Up* (Vertical)	0.1017	0.096	0.175	1.000	0.484
*H*0 (North)	0.5	0.638	0.765	0.955	0.817
*H*1 (East)	0.5	0.618	0.75	0.953	0.796
*Up* (Vertical)	0.5	0.113	0.204	1.000	0.484

**Table 6:** Event–station-level fusion performance of the proposed CNN–LSTM model on the real GNSS test dataset. Operating Point (OP) refers to either the fixed global threshold (τ = 0.5) or the validation-optimized val-bestF1 thresholds (selected by maximizing event–station-level F1 on aggregated event–station scores).

Fusion Rule	OP	Precision	F1	Recall	PR-AUC	ROC-AUC
Vote (2 of 3)	τ = 0.5	0.699	0.800	0.936	0.834	0.827
Vote (2 of 3)	val-bestF1	0.631	0.774	1.000	0.815	0.788

**Table 7:** Frame-level performance metrics of the proposed CNN–LSTM model on the real GNSS test dataset.

Channels	Decision Threshold	Precision	F1	Recall	PR-AUC
*H*0 (North)	0.4485	0.515	0.595	0.703	0.628
*H*1 (East)	0.4190	0.474	0.572	0.720	0.603
*Up* (Vertical)	0.4813	0.095	0.171	0.865	0.315
*H*0 (North)	0.5	0.535	0.600	0.684	0.628
*H*1 (East)	0.5	0.508	0.585	0.688	0.603
*Up* (Vertical)	0.5	0.096	0.174	0.859	0.315

**Table 8:** Channel-wise MMD (RBF kernel) between training (train + validation) and test distributions (95% CI).

GNSS Channels	$N_{VAL}$	$N_{TEST}$	MMD	Confidence Lower	Interval 95% Upper
*H*0	14,746	2966	0.1052	0.1001	0.1110
*H*1	14,746	2966	0.0939	0.0898	0.0989
*UP*	14,746	2966	0.0663	0.0622	0.0709

**Table 9:** Mean absolute SHAP values for validation and test sets, illustrating how the model’s attributional behavior changes under distributional shift.

	Mean Absolute SHAP		Attribution	Attribution Inflation
GNSS Channels	Validation µ(val)	Test µ(val)	Shift (Δ) (µ(test) − µ(val))	Ratio (µ(test)/µ(val))
*H*0	0.000852	0.002587	0.001735	3.04
*H*1	0.001067	0.002821	0.001754	2.64
*UP*	0.001165	0.003075	0.001910	2.64

**Table 10:** Quantitative comparison of the proposed CNN–LSTM hybrid model and baseline approaches on the real GNSS test dataset under the event–station evaluation setting.

Model	Threshold	Precision	Recall	F1	PR-AUC
CNN-LSTM Hybrid	0.5	0.6993	0.9357	0.8004	0.8339
LSTM-only	0.5	0.7604	0.8843	0.8177	0.8469
CNN-only	0.5	0.7167	0.8940	0.7956	0.8326
RFM [11]	0.372	0.6042	1.0000	0.7532	0.9475

## Data Availability

The datasets analyzed in this study were obtained from publicly available third-party resources. The SNIVEL software used for TDCP-based velocity processing is openly available at https://github.com/crowellbw/SNIVEL (accessed 5 January 2026) [30]. The 5 Hz GNSS velocity time series for real earthquake events used in this study are available via Zenodo (DOI: 10.5281/zenodo.6588601) [18]. Labeled 5 Hz GNSS velocity samples and pseudo-synthetic samples are available via Zenodo (DOI: 10.5281/zenodo.7909327) [11]. The 5 Hz GNSS ambient data used for TDCP processing are available from the GAGE GNSS archive (EarthScope; previously UNA-VCO) in RINEX (v2.11) format at https://data.unavco.org/archive/gnss/highrate/5-Hz/rinex/ (accessed 5 January 2026).

## Supplementary Materials

The following supporting information can be downloaded at https://www.mdpi.com/article/10.3390/s26020519/s1: Table S1: Ablation results for alternative fusion rules (any-channel and weighted) at the two operating points (τ = 0.5 and val-bestF1) on the real GNSS test dataset. Table S2: Ablation of event-score aggregation modes (max, mean, p90) on the real GNSS test set at the event–station level using the vote-based (2-of-3) fusion rule. Because aggregation changes the score scale, mode-specific validation-optimized best-F1 thresholds are used instead of a fixed threshold (τ = 0.5).
