# Supplementary material for: A Hybrid CNN-LSTM Architecture for Seismic Event Detection Using High-Rate GNSS Velocity Time Series
Source: Sensors (Basel). 2026 Jan 13;26(2):519. doi: 10.3390/s26020519 (PMC12845801; doi:10.3390/s26020519)
Supplement: Supplementary file 1 [file sensors-26-00519-s001.zip › sensors-4019982-supplementary.pdf]

Supplementary Materials

Table S1. Ablation results for alternative fusion rules (any-channel and weighted) at the two operating points (τ = 0.5 and val-bestF1) on the real GNSS test dataset.

Fusion Rule	OP	Precision	F1	Recall	TN	FP	FN	TP	FPR
Vote (2 of 3)	τ = 0.5	0.699	0.800	0.936	1219	502	80	1165	0.292
	val-bestF1	0.631	0.774	1.000	992	729	0	1245	0.423
Weighted	τ = 0.5	0.699	0.800	0.936	1120	501	80	1165	0.291
	val-bestF1	0.631	0.774	1.000	992	729	0	1245	0.424
Any	τ = 0.5	0.699	0.801	0.940	1216	505	75	1170	0.293
	val-bestF1	0.631	0.774	1.000	992	729	0	1245	0.424

Table S2. Ablation of event-score aggregation modes (max, mean, p90) on the real GNSS test set at the event–station level using the vote-based (2-of-3) fusion rule. Because aggregation changes the score scale, mode-specific validation-optimized best-F1 thresholds are used instead of a fixed threshold (τ = 0.5).

Aggregation Mode	Precision	F1	Recall	PR-AUC	ROC-AUC	FPR
max	0.631	0.774	1.000	0.815	0.788	0.423
mean	0.633	0.775	0.998	0.816	0.790	0.418
p90	0.634	0.775	0.998	0.816	0.790	0.417
